# Effect of diazepam on sociability of rats submitted to neonatal seizures

**DOI:** 10.1016/j.dib.2016.03.029

**Published:** 2016-03-12

**Authors:** Ingrid Stanize Leite, Adelissandra S.S. Castelhano, Roberta M. Cysneiros

**Affiliations:** Developmental Disabilities Graduate Program, Laboratory of Neurobiology, Mackenzie Presbyterian University, Rua da Consolação, 930. Prédio 28, CEP 01302-907 São Paulo, SP, Brazil

**Keywords:** Neonatal seizures, Pilocarpine, Diazepam, Anxiety, Sociability

## Abstract

*Status epilepticus* (SE), an acute condition characterized by repetitive or ongoing seizures activity, may produce long-term deleterious consequences. Previous data demonstrated that Wistar rats subjected to neonatal SE displayed autistic behavior, characterized by social play impairment, low preference by novelty, deficit in social discrimination; anxiety related behavior and stereotyped behavior with no changes in locomotor activity (doi: http://dx.doi.org/10.1007/s00702-010-0460-1, doi: http://dx.doi.org/10.3389/fnbeh.2013.00036, doi: http://dx.doi.org/10.1007/s00702-014-1291-2[1], [2], [3]). Taking into account the bi-directional relationship between the state of anxiety and social interaction (doi: http://dx.doi.org/10.1007/s10567-009-0062-3[4]), we evaluated the impact of the state of anxiety on social interaction. Male Wistar rats at postnatal day 9 were subjected to pilocarpine-induced neonatal SE (380 mg/kg, ip) and the controls received 0.9% saline (0.1 ml/10 g). The groups received saline or diazepam (1.0 mg/kg) 45 min prior each behavioral testing that started from 60 days of postnatal life. In the open field, rats subjected to neonatal seizure exhibited less central zone activity as compared to animals treated with diazepam, with no changes in the total locomotor activity. In elevated plus maze, rats subjected to neonatal seizure and treated with diazepam exhibited higher locomotor activity and spent more time on the open arms as compared to untreated animals. In approach phase of sociability paradigm, animals subjected to neonatal seizures similarly to controls, regardless the treatment, spent more time with social stimulus as compared to non social stimulus. In social novelty phase of sociability paradigm, animals subjected to neonatal seizures differently of controls, regardless the treatment, spent similar time with familiar and novel stimulus.

**Specifications table**

**Table:** 

Subject area	*Biology*
More specific subject area	*Behavioral Neuroscience*
Type of data	*Figure*
How data was acquired	*Elevated plus maze*, *Open field*, *Three chamber box to sociability test*
Data format	*Analyzed data*
Experimental factors	*Males Wistar rats* (*PN9*) *were subject to pilocarpine-induced Status epilepticus* (*380 mg*/*kg, ip*) *and control animals received saline*.
Experimental features	*Young adults rats* (*PN60-80*) *subject to neonatal SE were probed for open field, elevated plus maze and sociability paradigms 45 min after saline or diazepam* (*1 mg/kg, i.p.*) *administration*.
Data source location	*São Paulo*, *Brazil*
Data accessibility	*Data provided in this article*

**Value of the data**

• Neonatal seizures in rats decreased the central zone activity but not the total locomotor activity in the open field.
• Treatment with diazepam increased the central zone activity of rats submitted to neonatal seizures.
• Neonatal seizures in rats decreased the time spent on the open arms and the locomotor activity in elevated plus maze, which was reverted by pretreatment with diazepam.
• In approach phase of sociability paradigm, animals subjected to neonatal seizures similarly to controls spent more time with social stimulus as compared to non social stimulus.
• In social novelty phase of sociability paradigm, animals subjected to neonatal seizures spent similar time with familiar and novel stimulus, which was not affected by treatment with diazepam.

## 1 Data

**Fig. 1:**
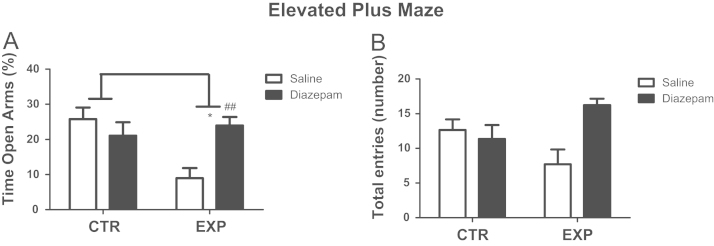
Percentage of time spent in the open arms (A) and total entries in both arms (B) are shown as mean±standard error. EXP animals spent less time on the open arms as compared to CTR group (*p=0.032) which was reserved by treatment with diazepam (##*p*<0.01).

**Fig. 2:**
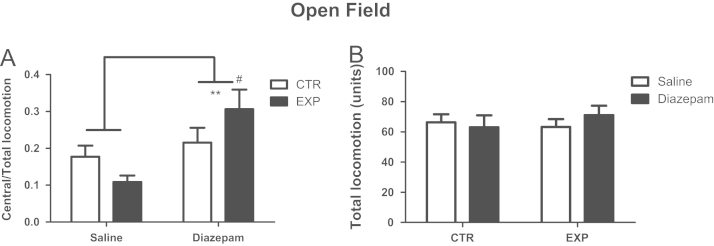
Ratio of central/total locomotion (A) and total locomotion (B) are shown as mean±standard error. EXP treated with saline exhibited less activity in central zone of the apparatus which was reversed by treatment with diazepam. Total locomotion did not differ between groups nor treatment. **p<0.001 (effect of treatment) and #*p*<0.05 (difference between experimental animals treated with saline and diazepam).

In elevated plus maze, for time spent in the open arms, it was noted significant effects of interaction between factors treatment×groups (*F*(1,48)=9.873; *P*=0.003) and difference between groups (*F*(1,48)=4.859; *P*=0.032), for entries in both arms, it was noted effect of interaction between treatment×group (*F*(1,48)=8.19; *P*=0.0062) and the effect of treatment (*F*(1,48)=4.412; *P*=0.0062). Experimental group spent less time on the open arms and decreased locomotor activity reversed by diazepam (Fig. 1). In open field, for ratio by central/total locomotion, it was noted effect of treatment (*F*(1,48)=7.9; *P*=0.0071), and central locomotor activity of experimental animals was increased by treatment with diazepam (*t*=3.37; *P*<0.05). Total locomotion was not affect by SE nor by treatment with diazepam (Fig. 2).

**Fig. 3:**
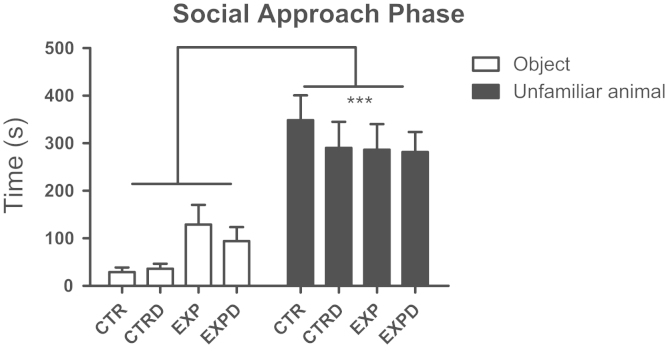
Time spent into compartments with object and unfamiliar rat is shown as mean±standard error. Both groups, regardless the treatment, showed a clear preference for the social stimulus. (^⁎⁎⁎^*P*=0.0001).

In social approach phase of the sociability test, both groups, regardless the treatment (*F*(1,48)=0.67; *P*=0.42), spent more time in the compartment with unfamiliar rat (*F*(1,48)=61.52; *P*=0.0001), (Fig. 3).

**Fig. 4:**
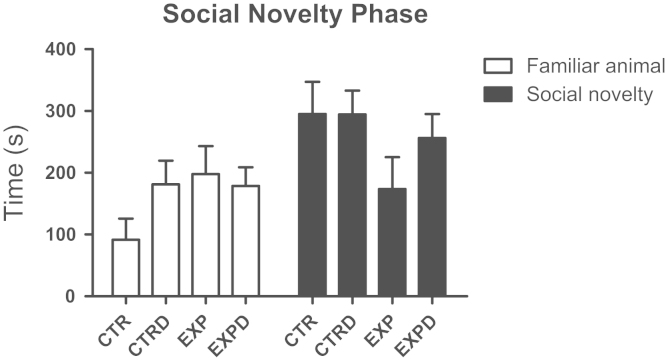
Time spent into compartments with familiar animal and social novelty is shown as mean±standard error. Control animals exhibited preference by social novelty and experimental animals displayed deficit in social discrimination unaffected by diazepam (*F*(1,48)=4.793; *P*=0.033).

It was noted a significant effect of compartment (*F*(1,48)=8.262; *P*=0.006) and interaction between compartment×groups (*F*(1,48)=4.793; *P*=0.033). Experimental group, regardless the treatment, did not discriminate between familiar and novel stimulus (Fig. 4).

## 2 Experimental design, materials and methods

All procedures were approved by Universidade Presbiteriana Mackenzie Ethical Committee (CEUA, 106/02/2014). Newly born Wistar male rats were maintained under controlled conditions (07:00–19:00 h, light/dark cycle; 22–24 °C) with their mothers. Pups׳ ages were determined from the day of birth (P0). Colonies were randomly assigned into different groups. All procedures were carried out in male rats.

## 3 ***Status epilepticus*** i**nduction**

SE is usually defined as continuous seizure activity lasting for 30 min or longer or intermittent seizures lasting 30 min or more from which the patient does not regain consciousness (Commission on Classification and Terminology of the International League Against Epilepsy – ILAE, 1989). The experimental group received pilocarpine 3.8% in saline (380 mg/kg, i.p), on P9 which corresponds to a full-term neonate [5], and the control group received saline solution (0.1 mL/10 g). SE started within 3–4 min following pilocarpine injection being characterized by continuous intense body tremor, scratching, clonic movements of forelimbs and head bobbing. Following cessation of SE (ca 4 h) animals returned to their mothers. At 21 days postnatal, 2–3 male animals of each litter were randomly chosen, housed together (4–5 animals per cage) and distributed in the following groups:

• Control group+saline (CTR): 12 rats that received saline injection.
• Control group+Diazepam (CTRD): 13 treated with diazepam (1 mg/kg, i.p).
• Experimental group+saline (EXP): 11 rats that received saline injection.
• Experimental group+diazepam (EXPD): 16 rats treated with diazepam (1 mg/kg, i.p).

The behavioral tests started from 60 days postnatal and were videotaped. The animals received diazepam or saline 45 min before tests. The animals were transferred to the testing room 60 min before each day session. All apparatus were cleaned with a 5% alcohol solution after each behavioral procedure. All behavioral tests were carried out in the same room with a controlled intensity of light (9 lx).

## 4 Elevated plus maze

The apparatus had two closed arms with walls 45 cm in height and two open arms 50 cm long (Insight Ltda., Brazil). The maze was elevated 50 cm from the floor. The animals were placed in the center of the maze with their nose pointing towards an open arm and they were allowed to freely explore the maze for 10 min. The sections were videotaped, and the time spent in both arms was recorded and expressed as percent of time in open arms [(Open arm/Open arm+Closed arm)×100].

## 5 Open field

The apparatus was a circular arena (100 cm diameter) enclosed by plain white walls and a floor divided into 12 zones, being 8 peripheral and 4 central (Insight Ltda., Brazil). Each animal was placed into the central area and observed for 10 min. The locomotor activity was expressed as the number of total lines crossed and by ratio of central to total locomotion.

## 6 Sociability

The social behavior apparatus was adapted by Ref. [6] from Ref. [7]. The apparatus was an acrylic rectangular box divided into three compartments of equal size (39 cm height×26 cm width×41 cm deep) by retractable doors. The sociability test, preceded by a habituation period in the apparatus, was divided in 3 sequential phases of 10 min each. During the habituation period, the test rat was placed in the middle chamber for 10 min with the retractable doors closed. Each of the two sides contained an identical empty wire cage. In the social approach phase, an unfamiliar rat was enclosed in one of the wire cages and the time spent in each compartment with objects or social stimulus was measured. In the social novelty phase, a new unfamiliar rat was enclosed into the wire cage in the opposite compartment and the time spent in each compartment was measured. It is important to mention that before the introduction of a social stimulus the test rat was trapped in the central chamber.

## 7 Statistical analysis

The data were expressed as mean±standard error. The sociability test was analyzed by Mixed ANOVA using compartment (object 1×object 2, object×unfamiliar rat or familiar rat versus social novelty) as within-subjects factor and groups (EXP versus CTR) and treatment (saline×diazepam) as between-subjects factor [8]. Significant effects were probed with post-hoc testing (Bonferroni). The Open Field׳s and Elevated Plus Maze׳s parameters were analyzed by Two-way ANOVA, using Bonferroni for post-hoc testing. *P* values of 0.05 or less were considered significant.
